# Correction: Dilp-2–mediated PI3-kinase activation coordinates reactivation of quiescent neuroblasts with growth of their glial stem cell niche

**DOI:** 10.1371/journal.pbio.3001438

**Published:** 2021-10-19

**Authors:** Xin Yuan, Conor W. Sipe, Miyuki Suzawa, Michelle L. Bland, Sarah E. Siegrist

## Notice of Republication

This article was republished on August 9, 2021 to address reporting issues that were noted after the initial version of the article was posted online. Specifically, it was noted that the *PLOS Biology* article did not adequately discuss or cite relevant literature. In the updated and republished version, the authors provide additional discussion of prior studies and clearly delineate how their *PLOS Biology* study built upon previous findings and made novel contributions to this research area. The originally published, uncorrected article and the republished, corrected articles are provided here for reference.

## Supporting information

S1 File Originally published uncorrected article. (PDF)

S2 File Republished, corrected article. (PDF)
