# Supplementary figures and images for: Dilp-2–mediated PI3-kinase activation coordinates reactivation of quiescent neuroblasts with growth of their glial stem cell niche
Source: PLoS Biol. 2020 May 28;18(5):e3000721. doi: 10.1371/journal.pbio.3000721 (PMC7282672; doi:10.1371/journal.pbio.3000721)

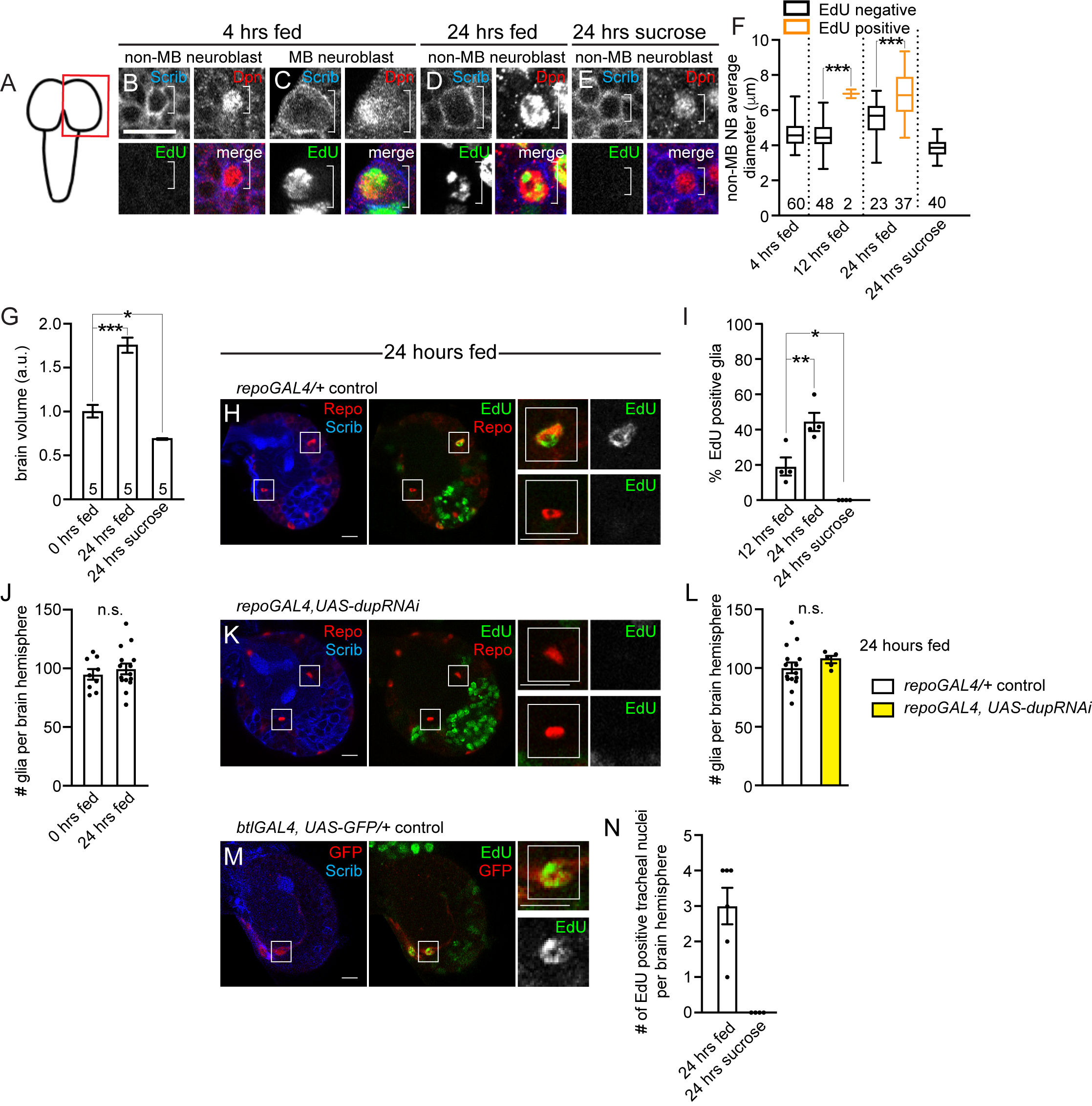

Supplement: S1 Fig — (A) Larval brain cartoon with red box indicating the brain region imaged in Fig 1 and all subsequent Figs. (B–E) Single confocal Z images of non-MB and MB NBs. Top and bottom left panels are single-channel grayscale images, with colored overlay in the bottom right. Molecular markers are denoted within panels and white brackets indicate NBs in this and all figures. (F) Box and whisker plots of NB diameter of EdU-negative (black) and EdU-positive (orange) NBs. The number of NBs analyzed is indicated below plots. Student two-tailed t test, ***p < 0.001. (G) Fold changes in brain volume per hemisphere in response to animal feeding (see Materials and Methods). Column numbers indicate number of brain hemispheres scored. Values are normalized to 0-hour–fed animals. (H) Single Z-plane of a brain hemisphere. Left and middle panels are color overlays of a control brain after feeding. High-magnification images of 2 Repo-positive glia are shown to the right, one EdU-positive and one EdU-negative (white boxes). (I) Quantification of EdU-positive glial cells per brain lobe in response to feeding. (J) Number of Repo-positive glial cells per brain hemisphere before and after animal feeding. (K) Single Z image of a brain hemisphere from a dupRNAi knockdown animal. Left and middle panels are color overlays with high magnification of 2 Repo-positive glia shown to the right (white boxes). (L) Quantification of glia number in dupRNAi knockdown animals compared to control. (M) Single Z image of a brain hemisphere. Left and middle panels are color overlays of control brain after feeding. A high-magnification image of a tracheal nucleus is shown to the right (white boxes). (N) Quantification of EdU in trachea from animals fed standard food or sucrose. (G,I) One-way ANOVA with Tukey post hoc analysis and (F,J,L,N) Student two-tailed t test, *p < 0.05, **p < 0.01, ***p < 0.001. Genotypes of panels listed in S2 Table and data listed in S1 Data. dupRNAi, double-parked RNA interference; EdU, 5- [file pbio.3000721.s001.tif]

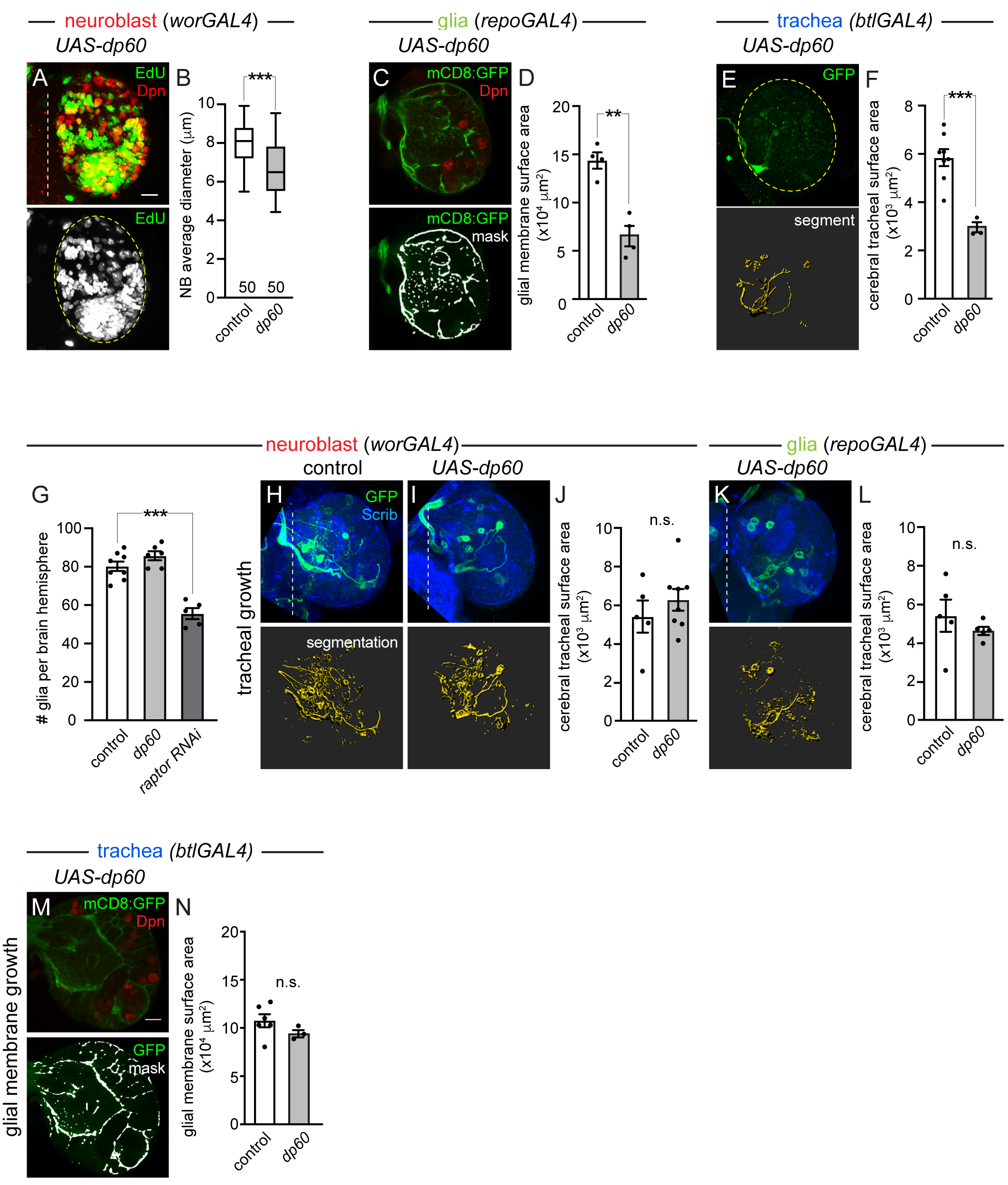

Supplement: S2 Fig — (A) Maximum intensity projection of a single brain hemisphere labeled with EdU and quantification of NB size from indicated genotypes (B). (C,M) Glial and (E,H,I,K) tracheal morphology with quantification of glial surface area (D,N) and tracheal surface area from indicated genotypes (F,J,L). Top panels are colored overlays, with bottom panels showing single-channel grayscale images (A), single channel and mask (C,M), or rendered maximum intensity projection with segmentation of trachea (E,H,I,K). Molecular markers are denoted within panels. (G) Quantification of glial number. (B,D,F,J,L,N) Student two-tailed t test, *p < 0.05, **p < 0.01,***p < 0.001, error bars, SEM. (G) One-way ANOVA with Tukey post hoc analysis. Genotypes of panels listed in S2 Table and data listed in S1 Data. EdU, 5-ethynyl-2′-deoxyuridine; NB, neuroblast; PI3-kinase, phosphoinositide 3-kinase. (TIF) [file pbio.3000721.s002.tif]

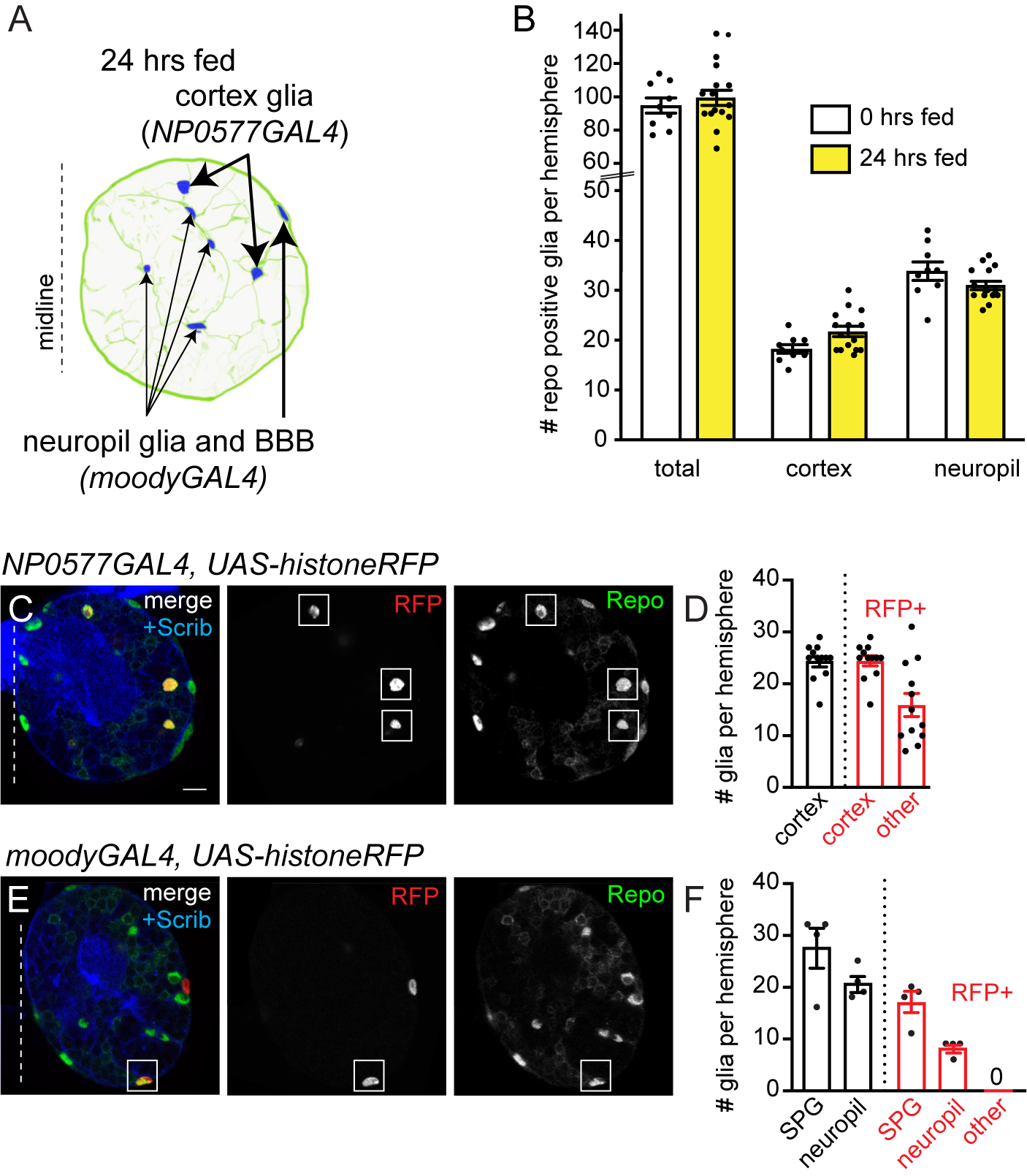

Supplement: S3 Fig — (A) Single Z image of a segmented brain hemisphere with glia subtypes and corresponding GAL4 lines. (B) Total number of glia subtypes before and after animal feeding. Glia type was identified based on location. Error bars, SEM. Black circles indicate single brain hemispheres. (C and E) Left panels, colored overlays of a single Z-plane from a brain hemisphere of the indicated genotype, with grayscale images on the right. Molecular markers are denoted within panels. Quantification of glial populations are shown in D and F. Red columns depict mean of histoneRFP-expressing glia, and black columns depict mean of glia identified based on position. Whites boxes indicate RFP-expressing cortex glia in (C) and SPG in (E). Genotypes of panels listed in S2 Table and data listed in S1 Data. RFP, red fluorescent protein; SPG, subperineurial glia. (TIF) [file pbio.3000721.s003.tif]

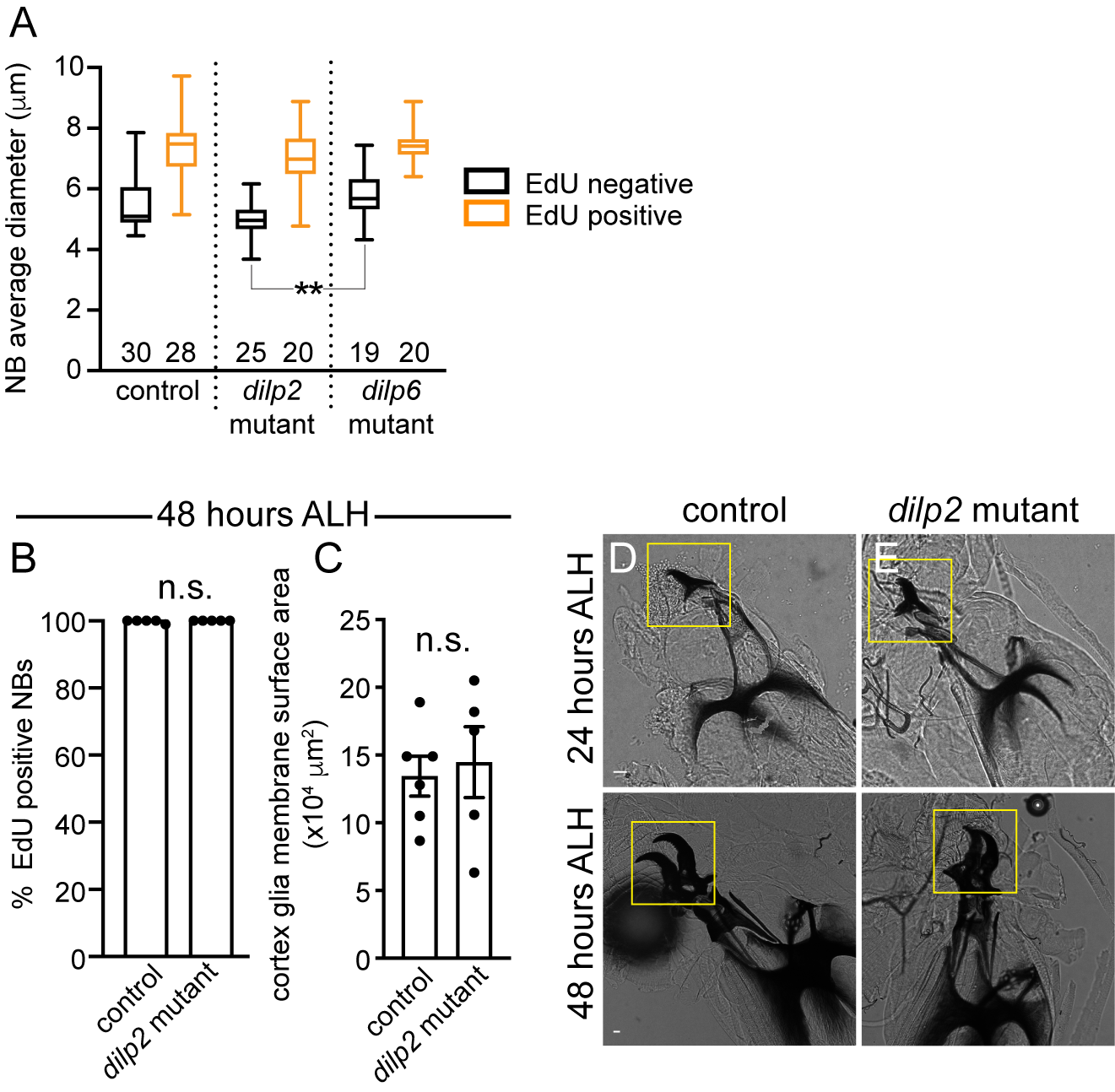

Supplement: S4 Fig — (A) Box plots of NB diameter of EdU-negative (black) versus EdU-positive (orange) NBs. Column numbers indicate number of NBs analyzed. (B,C) Quantification of EdU-positive neuroblasts (B) and cortex glial membrane surface (C) area after 48 hours of feeding of indicated genotypes. (D,E) Mouth hook morphology of control and dilp2 mutants at indicated times. (A–C) Student two-tailed t test, **p < 0.01. Genotypes of panels listed in S2 Table and data listed in S1 Data. Dilp, Drosophila insulin-like peptide; EdU, 5-ethynyl-2′-deoxyuridine; NB, neuroblast. (TIF) [file pbio.3000721.s004.tif]

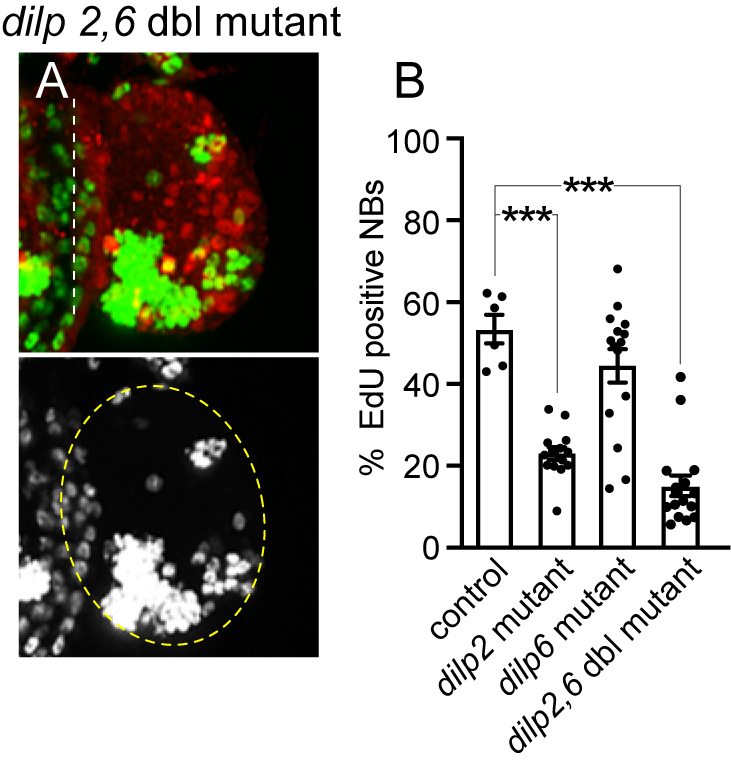

Supplement: S5 Fig — (A,B) EdU-positive NBs after 24 hours of feeding in dilp2, dilp6 double mutants. (A) Maximum intensity projection of a brain hemisphere. Top panel is a colored overlay (red, Dpn; green, EdU), and the bottom panel is a single-channel grayscale image (EdU) below with quantification in (B). Brain hemispheres are outlined, and the dotted vertical line indicates the midline. (B) One-way ANOVA with Tukey post hoc analysis. ***p < 0.001. Genotypes of panels listed in S2 Table and data listed in S1 Data. Dilp, Drosophila insulin-like peptide; Dpn, Deadpan; EdU, 5-ethynyl-2′-deoxyuridine; NB, neuroblast. (TIF) [file pbio.3000721.s005.tif]
