# Supplementary material for: Dilp-2–mediated PI3-kinase activation coordinates reactivation of quiescent neuroblasts with growth of their glial stem cell niche
Source: PLoS Biol. 2020 May 28;18(5):e3000721. doi: 10.1371/journal.pbio.3000721 (PMC7282672; doi:10.1371/journal.pbio.3000721)
Supplement: S1 Table — (DOCX) [file pbio.3000721.s006.docx]

|  | **Glia expression** | | | | other expression |
| --- | --- | --- | --- | --- | --- |
| **GAL 4 line** | cortex glia | SPG | neuropil glia | astrocytes |  |
| *repoGAL4* | +++ | +++ | +++ | +++ | none |
| *NP0577Gal4* | +++ | + | + | - | few neurons |
| *NP2222Gal4* | ++ | - | - | - | trachea and IPCs |
| *moodyGal4* | - | ++ | + | - | NA |
| *spinGal4* | - | ++ | - | - | few neurons and semi-viable |
| *wunGal4* | +++ | - | +++ | +++ | NA |

+++ expressed in all glia

++ expressed in some glia

+ expressed in few glia

- expressed in no glia

NA, not assayed
