# Supplementary material for: Dilp-2–mediated PI3-kinase activation coordinates reactivation of quiescent neuroblasts with growth of their glial stem cell niche
Source: PLoS Biol. 2020 May 28;18(5):e3000721. doi: 10.1371/journal.pbio.3000721 (PMC7282672; doi:10.1371/journal.pbio.3000721)
Supplement: S2 Table — (DOCX) [file pbio.3000721.s007.docx]

| **FIGURE** | **GENOTYPE** | | | |
| --- | --- | --- | --- | --- |
| Figure 1A-F | *repoGal4/+ (OregonR)* | |  | |
| Figure 1G-L | *UAS-mCD8GFP, repoGal4/+ (OregonR)* | |  | |
| Figure 1M-R | *btlGal4, UAS-GFP/+ (OregonR)* | |  | |
| Figure 2B,E | *repoQF2, QUAS-mCD8GFP/+ (OregonR)* | | control | |
| Figure 2C,E | *worGal4, UAS-dp60/repoQF2,QUAS-mCD8GFP* | |  | |
| Figure 2D,E | *worGal4; repoQF2,QUAS-mCD8GFP/UAS-raptorRNAi* | |  | |
| Figure 2F,H,I | *repoGal4/+ (OregonR)* | | control | |
| Figure 2G,H,I | *repoGal4/UAS-dp60* | |  | |
| Figure 2J,K | *btlGal4, UAS-GFP/UAS-dp60* | |  | |
| Figure 2K | *btlGal4, UAS-GFP/+ (OregonR)* | | control | |
| Figure 3A,B,D | *NP0577Gal4, UAS-mCD8GFP* | |  | |
| Figure 3C,D | *NP0577Gal4, UAS-mCD8GFP, UAS-dp60* | |  | |
| Figure 3E,F,H | *moodyGal4, UAS-mCD8GFP* | |  | |
| Figure 3G,H | *moodyGal4, UAS-mCD8GFP/UAS-dp60* | |  | |
| Figure 4A,D,E | *NP0577Gal4/+(OregonR)* | | control | |
| Figure 4B,D,E | *NP0577Gal4/UAS-dp60* | |  | |
| Figure 4C,D,E | *NP0577Gal4/UAS-grim* | |  | |
| Figure 4F,H | *moodyGal4/+(OregonR)* | | control | |
| Figure 4G,H | *moodyGal4/UAS-dp60* | |  | |
| Figure 4J | *NP0577GAL4, UAS-histoneRFP/UAS-grim* | |  | |
| Figure 4K | *NP0577GAL4, UAS-mCD8GFP/UAS-grim* | |  | |
| Figure 5A | *OregonR/w^1118^ and dilp1^1^/dilp1^1^ and dilp2^1^/dilp2^1^ and dilp3^1^/dilp3^1^ and dilp4^1^/dilp4^1^ and dilp5^1^*/*dilp5^1^* and *dilp6^68^/dilp6^68^* and *dilp7^1^/dilp7^1^ and dilp2^1^/Df(3L)Ilp2-3* | | | |
| Figure 5B | *OregonR/w^1118^* | | | control |
| Figure 5C | *dilp2^1^/dilp2^1^* | | | |
| Figure 5D | *dilp6^68^/dilp6^68^* | | | |
| Figure 5E,G | *NP0577Gal4, UAS-mCD8GFP* | | | control |
| Figure 5F,G | *NP0577Gal4, dilp2^1^/UAS-mCD8GFP, dilp2^1^* | |  | |
| Figure 5G | *NP0577Gal4, Df(3L)Ilp2-3/UAS-mCD8GFP, dilp2^1^* | |  | |
| Figure 5H,J | *btlGal4, UAS-GFP/+ (OregonR)* | | | control |
| Figure 5I,J | *btlGal4, UAS-GFP, dilp2^1^/dilp2^1^* | |  | |
| Figure 6A,C | *repoQF2, QUAS-mCD8GFP/+ (OregonR)* | | | control |
| Figure 6B,C | *dilp6^68^; repoQF2, QUAS-mCD8GFP* | |  | |
| Figure 6D,F | *btlGal4, UAS-GFP/+ (OregonR)* | | | control |
| Figure 6E,F | *dilp6^68^; btlGal4, UAS-GFP* | |  | |
| Figure 6G,H | *Oregon R* and *dilp2^1^/dilp2^1^* | |  | |
| Figure 6I | *dilp2^HA^* and *dilp6^HF^* | |  | |
| Figure 6J | *dilp6^HF^ and dilp6^HF^;dilp2^1^/dilp2^1^* | |  | |
| Figure 6K | *UAS-dilp2, dilp2^1^/dilp2^1^*  *dilp2GAL4, dilp2^1^/ UAS-dilp2, dilp2^1^*  *moodyGAL4, dilp2^1^/UAS-dilp2, dilp2^1^*  *NP0577GAL4, dilp2^1^/UAS-dilp2, dilp2^1^*  *dilp2GAL4, dilp2^1^/ UAS-dilp6, dilp2^1^* | |  | |
| Figure 7A | *pcnaGFP; repoGAL4, UAS-mCD8RFP* | |  | |
| Figure 7B-G | *UAS-mCD8GFP, repoGal4/+ (OregonR)* | |  | |
| Figure 7H,K | *UAS-mCD8GFP, repoGal4/UAS-dp60* | |  | |
|  | *UAS-mCD8GFP, repoGal4/+ (OregonR)* | | control | |
| Figure 7I,K | *NP0577Gal4, UAS-mCD8GFP/+ (OregonR)* | | control | |
|  | *NP0577Gal4, dilp2^1^/UAS-mCD8GFP, dilp2^1^* | |  | |
| Figure 7J,K | *repoQF2, QUAS-mCD8GFP/+ (OregonR)* | | control | |
|  | *worGal4, UAS-dp60/repoQF2, QUAS-mCD8GFP* | |  | |
| S1 Figure B-J | *repoGal4/+ (OregonR)* | |  | |
| S1 Figure K | *repoGal4/UAS-dupRNAi* | |  | |
| S1 Figure M,N | *btlGal4, UAS-GFP/+ (OregonR)* | |  | |
| S2 Figure A,B | *worGAL4/UAS-dp60* | |  | |
|  | *worGal4/+ (OregonR)* | | control | |
| S2 Figure C,D | *UAS-mCD8GFP, repoGal4/+ (OregonR)* | | control | |
|  | *UAS-mCD8GFP, repoGal4/UAS-dp60* | |  | |
| S2 Figure E,F | *btlGal4, UAS-GFP/+ (OregonR)* | | control | |
|  | *btlGal4, UAS-GFP/UAS-dp60* | |  | |
| S2 Figure H,I,J | *btlLexA, LexAOP-mCD8GFP* | | control | |
|  | *worGal4/UAS-dp60; btlLexA, LexAOP-mCD8GFP* | |  | |
| S2 Figure K,L | *btlLexA, LexAOP-mCD8GFP* | | control | |
|  | *UAS-dp60; repoGal4/btlLexA, LexAOP-mCD8GFP* | |  | |
| S2 Figure M,N | *repoQF2, QUAS-mCD8GFP/+ (OregonR)* | | control | |
|  | *btlGAL4/UAS-dp60, repoQF2, QUAS-mCD8GFP* | |  | |
| S3 Figure B | *repoGal4/+ (OregonR)* | |  | |
| S3 Figure C,D | *NP0577Gal4/UAS-histoneRFP* | |  | |
| S3 Figure E,F | *moodyGal4/UAS-histoneRFP* | |  | |
| S5 Figure A-E | *OregonR/w^1118^* | control | | |
|  | *dilp2^1^/dilp2^1^ and* *dilp6^68^/dilp6^68^* |  | | |
| S6 Figure A | *dilp6^68^/dilp6^68^; dilp2^1^/dilp2^1^* | |  | |
