# Supplementary material for: Dilp-2–mediated PI3-kinase activation coordinates reactivation of quiescent neuroblasts with growth of their glial stem cell niche
Source: PLoS Biol. 2020 May 28;18(5):e3000721. doi: 10.1371/journal.pbio.3000721 (PMC7282672; doi:10.1371/journal.pbio.3000721)
Supplement: S3 Table — (DOCX) [file pbio.3000721.s008.docx]

| **DROSOPHILA STOCK LIST AND SOURCE** | |
| --- | --- |
| OregonR | Bloomington Drosophila Stock Center #5 |
| *worGal4* | (57) |
| *UAS-mCD8GFP* on II | Bloomington Drosophila Stock Center #5137 |
| *UAS-mCD8GFP* on III | Bloomington Drosophila Stock Center #5130 |
| *repoGal4* | Bloomington Drosophila Stock Center #7415 |
| *btlGal4, UAS-GFP* | Bloomington Drosophila Stock Center #8807 |
| *btlGal4* on III | Bloomington Drosophila Stock Center #78328 |
| *UAS-dp60* | (30) |
| *repoQF2* | Bloomington Drosophila Stock Center #66477 |
| *QUAS-mCD8GFP* | Bloomington Drosophila Stock Center #30003 |
| *btlLexA* | Bloomington Drosophila Stock Center #66620 |
| *LexAOP-mCD8GFP* | Bloomington Drosophila Stock Center #32203 |
| *NP0577Gal4* | Kyoto Stock Center #112228 |
| *UAS-histoneRFP* | (58) |
| *pcnaGFP* | (59) |
| *moodyGal4* on II | (60) |
| *moodyGal4* on III | (60) |
| *UAS-grim* | isolated from Bloomington Drosophila Stock Center #52016 |
| *UAS-Pten* | Bloomington Drosophila Stock Center #82170 |
| *w1118* | Bloomington Drosophila Stock Center #3605 |
| *dilp1^1^* | (45) |
| *dilp2^1^* | (45) |
| *dilp3^1^* | (45) |
| *dilp4^1^* | (45) |
| *dilp5^1^* | (45) |
| *dilp6^68^* | (45) |
| *dilp7^1^* | (45) |
| *Df(3L)dilp2,3* | Bloomington Drosophila Stock Center #30888 |
| *dilp6HF* | (56) |
| *dilp2HF* | (61) |
| *dilp2GAL4* on II | Bloomington Drosophila Stock Center #37516 |
| *UAS-dilp2* on II | (43) |
| *UAS-dilp6* on III | (43) |
| *UAS-dupRNAi* | Bloomington Drosophila Stock Center #29562 |
| *UAS-raptorRNAi* | Bloomington Drosophila Stock Center #34814 |
| *UAS-mCD8RFP* | Bloomington Drosophila Stock Center |
| *NP2222GAL4* | Kyoto Stock Center #112830 |
| *spinGAL4* | Kyoto Stock Center #112853 |
| *wunGAL4* | Kyoto Stock Center #103953 |

1. Boulan L, Milan M, Leopold P. The Systemic Control of Growth. Cold Spring Harbor perspectives in biology. 2015;7(12): a019117.

2. Yamanaka N, Rewitz KF, O'Connor MB. Ecdysone control of developmental transitions: lessons from *Drosophila* research. Annu Rev Entomol. 2013;58: 497-516.

3. Hariharan IK. Organ Size Control: Lessons from *Drosophila*. Dev Cell. 2015;34(3): 255-65.

4. Koyama T, Mirth CK. Unravelling the diversity of mechanisms through which nutrition regulates body size in insects. Curr Opin Insect Sci. 2018;25: 1-8.

5. Danielsen ET, Moeller ME, Rewitz KF. Nutrient signaling and developmental timing of maturation. Curr Top Dev Biol. 2013;105: 37-67.

6. Nijhout HF, Riddiford LM, Mirth C, Shingleton AW, Suzuki Y, Callier V. The developmental control of size in insects. Wiley Interdiscip Rev Dev Biol. 2014;3(1): 113-34.

7. Chantranupong L, Wolfson RL, Sabatini DM. Nutrient-sensing mechanisms across evolution. Cell. 2015;161(1): 67-83.

8. del Valle Rodriguez A, Didiano D, Desplan C. Power tools for gene expression and clonal analysis in *Drosophila*. Nat Methods. 2011;9(1): 47-55.

9. Engelman JA, Luo J, Cantley LC. The evolution of phosphatidylinositol 3-kinases as regulators of growth and metabolism. Nat Rev Genet. 2006;7(8): 606-19.

10. Alamy M, Bengelloun WA. Malnutrition and brain development: an analysis of the effects of inadequate diet during different stages of life in rat. Neurosci Biobehav Rev. 2012;36(6): 1463-80.

11. Semba RD, Shardell M, Sakr Ashour FA, Moaddel R, Trehan I, Maleta KM, et al. Child Stunting is Associated with Low Circulating Essential Amino Acids. EBioMedicine. 2016;6: 246-52.

12. Semba RD, Zhang P, Gonzalez-Freire M, Moaddel R, Trehan I, Maleta KM, et al. The association of serum choline with linear growth failure in young children from rural Malawi. Am J Clin Nutr. 2016;104(1): 191-7.

13. Rafalski VA, Brunet A. Energy metabolism in adult neural stem cell fate. Prog Neurobiol. 2011;93(2): 182-203.

14. Ziegler AN, Levison SW, Wood TL. Insulin and IGF receptor signalling in neural-stem-cell homeostasis. Nat Rev Endocrinol. 2015;11(3): 161-70.

15. Colombani J, Raisin S, Pantalacci S, Radimerski T, Montagne J, Leopold P. A nutrient sensor mechanism controls *Drosophila* growth. Cell. 2003;114(6): 739-49.

16. Geminard C, Rulifson EJ, Leopold P. Remote control of insulin secretion by fat cells in *Drosophila*. Cell Metab. 2009;10(3): 199-207.

17. Britton JS, Edgar BA. Environmental control of the cell cycle in *Drosophila*: nutrition activates mitotic and endoreplicative cells by distinct mechanisms. Development. 1998;125(11): 2149-58.

18. Rajan A, Perrimon N. *Drosophila* cytokine unpaired 2 regulates physiological homeostasis by remotely controlling insulin secretion. Cell. 2012;151(1): 123-37.

19. Koyama T, Mirth CK. Growth-Blocking Peptides As Nutrition-Sensitive Signals for Insulin Secretion and Body Size Regulation. PLoS Biol. 2016;14(2): e1002392.

20. Delanoue R, Meschi E, Agrawal N, Mauri A, Tsatskis Y, McNeill H, et al. *Drosophila* insulin release is triggered by adipose Stunted ligand to brain Methuselah receptor. Science. 2016;353(6307): 1553-6.

21. Sousa-Nunes R, Yee LL, Gould AP. Fat cells reactivate quiescent neuroblasts via TOR and glial insulin relays in *Drosophila*. Nature. 2011;471(7339): 508-12.

22. Chell JM, Brand AH. Nutrition-responsive glia control exit of neural stem cells from quiescence. Cell. 2010;143(7): 1161-73.

23. Speder P, Brand AH. Systemic and local cues drive neural stem cell niche remodelling during neurogenesis in *Drosophila*. Elife. 2018;7: e30413.

24. Scadden DT. Nice neighborhood: emerging concepts of the stem cell niche. Cell. 2014;157(1): 41-50.

25. Lin S, Marin EC, Yang CP, Kao CF, Apenteng BA, Huang Y, et al. Extremes of lineage plasticity in the *Drosophila* brain. Curr Biol. 2013;23(19): 1908-13.

26. Sipe CW, Siegrist SE. Eyeless uncouples mushroom body neuroblast proliferation from dietary amino acids in *Drosophila*. Elife. 2017;6: e26343.

27. Unhavaithaya Y, Orr-Weaver TL. Polyploidization of glia in neural development links tissue growth to blood-brain barrier integrity. Genes & development. 2012;26(1): 31-6.

28. Pereanu W, Spindler S, Cruz L, Hartenstein V. Tracheal development in the *Drosophila* brain is constrained by glial cells. Dev Biol. 2007;302(1): 169-80.

29. Javaherian A, Kriegstein A. A stem cell niche for intermediate progenitor cells of the embryonic cortex. Cereb Cortex. 2009;19 Suppl 1: i70-7.

30. Weinkove D, Neufeld TP, Twardzik T, Waterfield MD, Leevers SJ. Regulation of imaginal disc cell size, cell number and organ size by *Drosophila* class I(A) phosphoinositide 3-kinase and its adaptor. Curr Biol. 1999;9(18): 1019-29.

31. Puig O, Marr MT, Ruhf ML, Tjian R. Control of cell number by *Drosophila* FOXO: downstream and feedback regulation of the insulin receptor pathway. Genes & development. 2003;17(16): 2006-20.

32. Siegrist SE, Haque NS, Chen CH, Hay BA, Hariharan IK. Inactivation of both Foxo and reaper promotes long-term adult neurogenesis in *Drosophila*. Curr Biol. 2010;20(7): 643-8.

33. Puig O, Tjian R. Transcriptional feedback control of insulin receptor by dFOXO/FOXO1. Genes & development. 2005;19(20): 2435-46.

34. Britton JS, Lockwood WK, Li L, Cohen SM, Edgar BA. *Drosophila's* insulin/PI3-kinase pathway coordinates cellular metabolism with nutritional conditions. Dev Cell. 2002;2(2): 239-49.

35. Ding R, Weynans K, Bossing T, Barros CS, Berger C. The Hippo signalling pathway maintains quiescence in *Drosophila* neural stem cells. Nat Commun. 2016;7: 10510.

36. Engler A, Rolando C, Giachino C, Saotome I, Erni A, Brien C, et al. Notch2 Signaling Maintains NSC Quiescence in the Murine Ventricular-Subventricular Zone. Cell Rep. 2018;22(4): 992-1002.

37. Read RD. Pvr receptor tyrosine kinase signaling promotes post-embryonic morphogenesis, and survival of glia and neural progenitor cells in *Drosophila*. Development. 2018;145(23): dev164285.

38. Inaba M, Buszczak M, Yamashita YM. Nanotubes mediate niche-stem-cell signalling in the *Drosophila* testis. Nature. 2015;523(7560): 329-32.

39. Sato M, Kornberg TB. FGF is an essential mitogen and chemoattractant for the air sacs of the *drosophila* tracheal system. Dev Cell. 2002;3(2): 195-207.

40. Shen Q, Wang Y, Kokovay E, Lin G, Chuang SM, Goderie SK, et al. Adult SVZ stem cells lie in a vascular niche: a quantitative analysis of niche cell-cell interactions. Cell Stem Cell. 2008;3(3): 289-300.

41. Tata M, Wall I, Joyce A, Vieira JM, Kessaris N, Ruhrberg C. Regulation of embryonic neurogenesis by germinal zone vasculature. Proc Natl Acad Sci U S A. 2016;113(47): 13414-9.

42. Brogiolo W, Stocker H, Ikeya T, Rintelen F, Fernandez R, Hafen E. An evolutionarily conserved function of the *Drosophila* insulin receptor and insulin-like peptides in growth control. Curr Biol. 2001;11(4): 213-21.

43. Ikeya T, Galic M, Belawat P, Nairz K, Hafen E. Nutrient-dependent expression of insulin-like peptides from neuroendocrine cells in the CNS contributes to growth regulation in *Drosophila*. Curr Biol. 2002;12(15): 1293-300.

44. Bader R, Sarraf-Zadeh L, Peters M, Moderau N, Stocker H, Kohler K, et al. The IGFBP7 homolog Imp-L2 promotes insulin signaling in distinct neurons of the *Drosophila* brain. J Cell Sci. 2013;126(Pt 12): 2571-6.

45. Gronke S, Clarke DF, Broughton S, Andrews TD, Partridge L. Molecular evolution and functional characterization of *Drosophila* insulin-like peptides. PLoS Genet. 2010;6(2): e1000857.

46. Colombani J, Andersen DS, Leopold P. Secreted peptide Dilp8 coordinates *Drosophila* tissue growth with developmental timing. Science. 2012;336(6081): 582-5.

47. Garelli A, Gontijo AM, Miguela V, Caparros E, Dominguez M. Imaginal discs secrete insulin-like peptide 8 to mediate plasticity of growth and maturation. Science. 2012;336(6081): 579-82.

48. Jaszczak JS, Wolpe JB, Bhandari R, Jaszczak RG, Halme A. Growth Coordination During *Drosophila* melanogaster Imaginal Disc Regeneration Is Mediated by Signaling Through the Relaxin Receptor Lgr3 in the Prothoracic Gland. Genetics. 2016;204(2): 703-9.

49. Colombani J, Andersen DS, Boulan L, Boone E, Romero N, Virolle V, et al. *Drosophila* Lgr3 Couples Organ Growth with Maturation and Ensures Developmental Stability. Curr Biol. 2015;25(20): 2723-9.

50. Garelli A, Heredia F, Casimiro AP, Macedo A, Nunes C, Garcez M, et al. Dilp8 requires the neuronal relaxin receptor Lgr3 to couple growth to developmental timing. Nat Commun. 2015;6: 8732.

51. Lim DA, Alvarez-Buylla A. The Adult Ventricular-Subventricular Zone (V-SVZ) and Olfactory Bulb (OB) Neurogenesis. Cold Spring Harbor perspectives in biology. 2016;8(5): a018820.

52. Rushing GV, Bollig MK, Ihrie RA. Heterogeneity of Neural Stem Cells in the Ventricular-Subventricular Zone. Adv Exp Med Biol. 2019;1169: 1-30.

53. Mirzadeh Z, Merkle FT, Soriano-Navarro M, Garcia-Verdugo JM, Alvarez-Buylla A. Neural stem cells confer unique pinwheel architecture to the ventricular surface in neurogenic regions of the adult brain. Cell Stem Cell. 2008;3(3): 265-78.

54. Silva-Vargas V, Maldonado-Soto AR, Mizrak D, Codega P, Doetsch F. Age-Dependent Niche Signals from the Choroid Plexus Regulate Adult Neural Stem Cells. Cell Stem Cell. 2016;19(5): 643-52.

55. Doyle SE, Pahl MC, Siller KH, Ardiff L, Siegrist SE. Neuroblast niche position is controlled by PI3-kinase dependent DE-Cadherin adhesion. Development. 2017; 144(5): 820-829.

56. Suzawa M, Muhammad NM, Joseph BS, Bland ML. The Toll Signaling Pathway Targets the Insulin-like Peptide Dilp6 to Inhibit Growth in *Drosophila*. Cell Rep. 2019;28(6): 1439-46 e5.

57. Albertson R, Doe CQ. Dlg, Scrib and Lgl regulate neuroblast cell size and mitotic spindle asymmetry. Nature cell biology. 2003;5(2): 166-70.

58. Langevin J, Le Borgne R, Rosenfeld F, Gho M, Schweisguth F, Bellaiche Y. Lethal giant larvae controls the localization of notch-signaling regulators numb, neuralized, and Sanpodo in *Drosophila* sensory-organ precursor cells. Curr Biol. 2005;15(10): 955-62.

59. Thacker SA, Bonnette PC, Duronio RJ. The contribution of E2F-regulated transcription to *Drosophila* PCNA gene function. Curr Biol. 2003;13(1): 53-8.

60. Schwabe T, Bainton RJ, Fetter RD, Heberlein U, Gaul U. GPCR signaling is required for blood-brain barrier formation in *drosophila*. Cell. 2005;123(1): 133-44.

61. Park S, Alfa RW, Topper SM, Kim GE, Kockel L, Kim SK. A genetic strategy to measure circulating *Drosophila* insulin reveals genes regulating insulin production and secretion. PLoS Genet. 2014;10(8): e1004555.
