# Supplementary material for: Dilp-2–mediated PI3-kinase activation coordinates reactivation of quiescent neuroblasts with growth of their glial stem cell niche
Source: PLoS Biol. 2020 May 28;18(5):e3000721. doi: 10.1371/journal.pbio.3000721 (PMC7282672; doi:10.1371/journal.pbio.3000721)
Supplement: Attachment — Submitted filename: reviewer response 3 04 20.docx [file pbio.3000721.s010.docx]

Please find attached our revised manuscript: **"Dilp-2 mediated PI3-kinase activation coordinates reactivation of quiescent neuroblasts with growth of their glial stem cell niche"** by Xin Yuan, Conor W. Sipe, Miyuki Suzawa, Michelle L. Bland, and Sarah E. Siegrist.

We very much appreciate the comments provided by the reviewers and their enthusiasm regarding the results.

In the revised version of the manuscript, we have rewritten sections of the text in order to better distinguish our work from work published previously and have included more detail regarding current knowledge of how Dilps and dietary nutrients regulate growth. In addition, we have moved some data to the supplement and have completed the following additional experiments as requested by reviewers. In brief:

1. **Experiment:** Neuroblast reactivation and glial membrane growth in animals transheterozygous for the *dilp2^1^* null allele over a small deficiency that removes *dilp2* and the neighboring *dilp3* locus.

**Result:**	Indistinguishable from *dilp2^1^* homozygotes (Figure 5A and G).

2. **Experiment:** Analysis of mouth hooks to determine whether *dilp2^1^* homozygous mutant animals are developmental delayed compared to wild type controls during the first 24 hours of larval development.

**Result:** *dilp2^1^* homozygous mutants are not developmentally delayed during early larval stages (S5D,E Figure).

3. **Experiment:** A series of *UAS-dilp2* rescue experiments in the *dilp2^1^* homozygous mutants.

**Result:** When *UAS-dilp2* is expressed in IPCs (*dilp2GAL4*) or in SPG (*moody GAL4*) in *dilp2^1^* homozygous mutants, nutrient-regulated neuroblast reactivation from developmental quiescence is rescued and partially rescued when *UAS-dilp2* is expressed in cortex glia (*NP0577GAL4*) (Figure 6K).

4. **Experiment:** A test to determine whether *UAS-dilp6* is sufficient to rescue neuroblast reactivation in *dilp2^1^* homozygous mutants.

**Result:** When *UAS-dilp6* is expressed in IPCs (*dilp2GAL4*) in *dilp2^1^* homozygous mutants, nutrient-regulated neuroblast reactivation from developmental quiescence is not rescued (Figure 6K). We chose to rescue in IPCs because this is where Dilp2 is normally expressed at this stage. Dilp2 is not expressed in SPG or other glia at this time.

5. **Experiment:** Assay neuroblast reactivation when PI3-kinase levels are reduced in both cortex glia and trachea.

**Result:** When PI3-kinase activity is reduced via simultaneous expression of *UAS-dp60* in both cortex glia (*NP0577GAL4*) and trachea (*btlGAL4*), there was a significant reduction in neuroblast EdU incorporation compared to animals expressing *UAS-dp60* in either cortex glia (*NP0577GAL4*) or trachea (*btlGAL4*), indicating impaired neuroblast reactivation (Figure 4I).

6. **Experiment:** Assay neuroblast reactivation and glial membrane growth in *dilp2^1^* homozygous mutant animals at 48 hours ALH.

**Result:** After 48 hours of animal feeding, *dilp2^1^* homozygous mutants are indistinguishable from controls based on the number of EdU positive neuroblasts and glial membrane surface area (S5B,C Figure).

Authors' point by point responses to **Reviewer 1's comments:**

Reviewer remarks:
**1.** **Consider splitting the quite long results section “Nutrient-dependent growth of neuroblasts, glia, and trachea requires cell autonomous and cell non-autonomous activation of PI3-kinase” into two sections, one for cell autonomous data and one for cell non-autonomous data. This is optional at author discretion.**

Thank you for this comment. We have rewritten part of the text in order to make it clearer and have split the very large figure into two.

**2. If the authors have data on knockdown of PI3K in both glia and trachea they should include it. These two cell types are likely functioning semi-redundantly in signaling to neuroblasts.**

We have carried out experiments to reduce PI3-kinase levels in both glia and trachea simultaneously. Indeed, we found that these two cell types are functioning in a semi-redundant manner to control neuroblast reactivation. This result is included in the manuscript in Figure 4I.

**3. Several glial subtype Gal4 lines characterized on page 8; why was the well-known astrocyte-specific alrm-Gal4 line excluded?** This was not included, because in our hands, we found alrm-GAL4 to be a sickly stock and we had a hard time collecting sufficient numbers of animals needed for experiments. We are in the process of outcrossing this line to improve viability.

**4. Page 8 second line from bottom. I think “compared” should be “comparable”**
We made that change.

**5. Page 10 second paragraph says “growth coordination between cortex glia and neuroblasts is Dilp-2 dependent” – which raises the question of what is the effect of creating a mismatch in growth coordination where neuroblasts and glia grow differently? Is there an effect on viability? Brain development?** This is a great question, an area of research we are currently investigating.

Authors' point by point responses to **Reviewer 2's comments:**

**This manuscript significantly overlapped with data previously reported (Pauline Speder and Andrea Brand, Elife, January 2018) in the following conclusions. 1) Neuroblast reactivation induces remodelling of cortex glia; 2) Nutrition-induced activation of PI3K/Akt drives the cortex glia to expand their membrane processes; 3) Dilps are required for development of cortex glia. One major claim of the current manuscript is the role of cortex glia in neuroblast reactivation, which contradicts with the previous conclusion that cortex glia has little impact on neuroblast reactivation (Elife 2018). The relevant findings on development/remodelling of cortex glia associated with neuroblast reactivation reported on Elife 2018 was wrongly referred to as SPG glia in the introduction on page 3 of this manuscript. The authors are advised to remove duplicated data and to provide the reasons underlying these different conclusions on the role of cortex glia in neuroblast reactivation.**

We thank the reviewer for this comment. We have rewritten the text in order to better differentiate our work from what is published and have worked to better acknowledge what is published from others. Major differences between our manuscript and work published previously (Pauline Speder and Andrea Brand, Elife, January 2018) include the following: (1) Time points analyzed (0 to 24 hours ALH **our work** versus 0 and 72 hours ALH **Speder and Brand, Elife 2018**), which is important because 0 to 24 hours is the time when neuroblasts initiate reactivation versus 72 hours, which is well after neuroblast reactivation and in fact at 72 hours ALH, neuroblasts are transitioning from early temporal factor expression (Imp) to later temporal factor expression (Syp) in response to systemic changes in ecdysone hormone levels. (2) Region of interest analyzed, brain (**our work**) versus ventral nerve cord (**Speder and Brand, Elife 2018**). This difference is significant because neuroblasts in the brain are not all located beneath the SPG and the tissue itself is sphere like, versus the ventral nerve cord, which is oblong and flat, and neuroblasts located in the VNC reportedly lie directly beneath the blood brain barrier. Beyond differences in tissue architecture, neuroblasts themselves are intrinsically different. In regards to your specific points:

**1) Neuroblast reactivation induces remodelling of cortex glia:**

In work published previously (Pauline Speder and Andrea Brand, Elife, January 2018), the authors assay "glial chamber" morphology at 72 hours ALH, which is well after the time when neuroblasts reactivate from developmental quiescence, even in neuroblasts over-expressing Pten. When levels of PI3-kinase activity are reduced in neuroblasts, reactivation from quiescence is delayed and once neuroblasts reactivate, proliferation rates are reduced. Thus, glial chamber impairment reported by Speder and Brand could be due to many things other than neuroblast reactivation, including generation of fewer neurons since neuroblast proliferation rates are reduced. In order to conclude that neuroblast reactivation induces remodeling of the cortex glia, the authors needed to assay glial morphology at earlier times, which they did not do.

In **our manuscript**, we quantify total glial membrane surface area at 0h, 4h, 12h, 16h, 20h and 24 hours ALH, which is the time when neuroblasts are indeed reactivating from developmental quiescence and report that increases in glial membrane surface area correlate with timing of neuroblast reactivation. Neuroblast reactivation and glial membrane growth are happening concurrently and when PI3-kinase levels are reduced in either cell type, growth of the other cell type is impaired. **This has not been established previously.** Specifically, we report that glial membrane surface area is reduced at 24 hours ALH when PI3-kinase activity is reduced in neuroblasts. Because we assay early (24 hours ALH) and not later (72 hours ALH), we can definitely conclude that this phenotype is due to defects in neuroblast reactivation and not a secondary less direct consequence due to differences in neuron number or sustained alteration of neuroblast proliferation rates.

**2) Nutrition-induced activation of PI3K/Akt drives the cortex glia to expand their membrane processes**

In work published previously (Pauline Speder and Andrea Brand, Elife, January 2018), the authors assay glial morphology at 72 hours ALH in fed animals and in animals raised on a sucrose only diet. They quantify Nrv2GFP fluorescence, however Nrv2GFP is not cortex glial specific, but labels other glial types (if not all), including subperineural glia. Again, because they assay only one time point (72 hours ALH), which is well after neuroblast reactivation, temporal resolution of glial growth in response to nutrition is lost.

In **our manuscript**, we use glia subtype specific GAL4 lines (NP0577GAL4 and moodyGAL4) to show that dietary nutrients and levels of PI3-kinase activation lead to increased growth of the different glial subtypes (cortex glia and SPG). We found that growth of both cortex glia and SPG is nutrient- and PI3-kinase-dependent. Whether growth of all glia is nutrient-regulated and PI3-kinase-dependent is certainly a possibility and a worthwhile area to investigate further. In addition, we show specifically that cortex glial contact with neuroblasts increases in response to dietary nutrients.

**3) Dilps are required for development of cortex glia.**

Using loss of function analysis, we demonstrate that Dilp2, but not Dilp6, is required for development of cortex glia. **This is the first report identifying a Dilp to be required for development of cortex glia.**

**One major claim of the current manuscript is the role of cortex glia in neuroblast reactivation, which contradicts with the previous conclusion that cortex glia has little impact on neuroblast reactivation (Elife 2018).**

In work published previously (Pauline Speder and Andrea Brand, Elife, January 2018), the authors report "we observed a small, but significant increase in PH3+ and PH3-EdU- neuroblasts at the expense of EdU+ neuroblasts" when PI3-kinase is reduced in cortex glia. This is consistent with our results, however it is unclear what precise developmental stage was analyzed by the authors. If it is assumed that the authors assay animals at 72 hours ALH as for the rest of their study, then our work (0-24 hours ALH) and their work is distinct for the same reasons as stated above.

In **our manuscript**, we genetically ablate almost all cortex glia (which we quantify based on double labeling with the glial specific antibody, Repo) and find that neuroblasts, while still all present, fail to reactivate after 24 hours of feeding. Moreover, we find that PI3-kinase activation in cortex glia is required for neuroblast reactivation in response to feeding. In our cortex glial genetic ablation experiments, animals do not survive past 36 hours ALH, therefore we cannot determine whether neuroblast reactivation is simply delayed as is the case with reduced PI3-kinase or whether neuroblasts never reactivate.

**The relevant findings on development/remodelling of cortex glia associated with neuroblast reactivation reported on Elife 2018 was wrongly referred to as SPG glia in the introduction on page 3 of this manuscript.**

We are unsure what the reviewer is referring to exactly, but have changed the original sentence "Subperineural (SPG) glia, a glial subtype that encapsulates the brain and ventral nerve cord to form a blood brain-like barrier, respond to the FDS by synthesizing and secreting Drosophila insulin-like peptide 6 (Dilp-6) locally within the brain (21-23)" to "Subperineural (SPG) glia, a glial subtype that forms part of the blood brain-like barrier, respond to the FDS by synthesizing and secreting Drosophila insulin-like peptide 6 (Dilp-6) locally within the brain (21-23)".

**The authors are advised to remove duplicated data and to provide the reasons underlying these different conclusions on the role of cortex glia in neuroblast reactivation.**

There is no data in our manuscript that has been duplicated or published previously. All previously published work has reported on neuroblast reactivation and glial biology in the VNC, and not in the brain hemispheres, and at a different developmental stage. The focus of our manuscript is the the first 24 hours time window after larval feeding begins which is when neuroblasts reactivate. For clarity and as requested, we have moved significant sections of data to supplement.

Authors' point by point responses to **Reviewer 3's comments:**

**Reviewer #3: In this paper Yuan and coworkers address how different cell types coordinate their growth in response to dietary nutrients. They use neuroblasts in the fly brain since these neural stem cells reactivate from quiescence in a nutrient dependent manner and are in close contact with other tissues in the niche (i.e. glia and trachea). The main claims of the paper are: 1)  that the coordinated growth between neuroblast and cortex glia is based on a bidirectional growth signaling between these two tissues. 2)  that dilp2 is required for neuroblast reactivation and cortex glia growth and hence the coordination of the two processes. 
The idea in first claim is not completely novel. Speder and Brand. eLife 2018;7:e30413 have shown that cortex glia growth in the niche is nutrient dependent and that it requires autonomous activation of PI3K . They also acknowledge the existence of another signal regulating cortex glia growth and link it to neuroblast reactivation. Yuan et al. main contribution to claim 1 is to propose that this signal is Dilp2 dependent PI3K activation in the neuroblast. If besides this contribution, the authors consider that their results add more to this claim, this was not evident in the text and they might want to emphasize any points that add to Speder and Brand findings.**

Reviewer 2 made a similar comment (see above). Speder and Brand claim that neuroblast reactivation is required for formation of the "glial chamber", a term they coin to refer to relative locations of neuroblasts, glia, and neurons within a confocal Z stack of the ventral nerve chord based on cell fluorescence intensities. However, Speder and Brand carry out their experiments at 72 hours ALH, which is well after the time when neuroblasts reactivate from developmental quiescence, even when PI3-kinase levels are reduced in neuroblasts. Furthermore, at this time (72 hours ALH) glial have undergone at least one round of cell division. Therefore, differences in the "glial chamber" between 3 day fed animals and 3 day sucrose animals could be due to differences in glia number. In our manuscript, we assay phenotypes within the first 24 hours ALH in the brain, which includes the time when neuroblasts are indeed reactivating from developmental quiescence and when glial number remains constant. In addition, Speder and Brand use Nrv2GFP, which is not expressed exclusively in cortex glia. While Speder and Brand do state that they use cyp4g15-GAL4, a "new" cortex glial driver, they do not characterize this line to show that GAL4 is expressed only in Repo positive glia located within the cortex. We observe that some of the authors conclusions--albeit aligning to some extent with our own--were drawn without presentation of supporting experiments. We have worked diligently to base our conclusions strictly on extensive sets of experiments, including detailed time series as well as critical loss of function and now rescue experiments. We believe that our detailed results provide a needed solid foundation to advance future research on the interplay of glia, neuroblasts, and trachea.

**The second claim is novel and is actually reflected in the title of the manuscript. Sousa-Nunes et al. 2011, Chell and Brand 2010, have shown that Dilps are involved in neuroblast reactivation (dilp2, 3, 5, 6) with some being expressed in glia (dilp3, 6), this paper addresses which dilp is required to coordinate the reactivation of neuroblasts and the growth of cortex glia. Through loss of function analyses they propose that dilp2 mediates neuroblast reactivation and cortex glia growth. They carry out a detailed analysis showing that an increase in glia-neuroblast membrane contacts increase takes place prior to neuroblast reactivation. These are interesting findings that would gain strength and benefit from additional experiments. Revision of the following major points is essential prior to publication: 
1.  The loss of function analysis of Dilps is done with null alleles generated though homologous recombination. It seems, based on the genotype provided, that they are analyzed in homozygous conditions. This has the risk of carrying additional 2nd site mutations in homozygosis. It would be reassuring to verify the Dilp2 results for neuroblast EdU incorporation and cortex glia growth with an additional allelic combination removing Dilp2 using two independent mutant chromosomes (dilp2/def or two dilp2 mutants from independent homologous recombination events).**

We have now analyzed EdU incorporation and cortex glial membrane growth in animals transheterozygous for the *dilp2^1^* null allele over a small deficiency that removes the *dilp2* and neighboring *dilp3* locus. Results were indistinguishable from *dilp2^1^* homozygotes, suggesting no 2nd site mutation causing phenotypes (Figure 5A and G).

**2.  Dilp2 null mutant has been reported to have a developmental delay. A time line experiment analyzing reduction in EdU incorporation and cortex glia growth at later time points than 24hrs of feeding  could distinguish whether the phenotypes observed are sustained in time or a consequence of the developmental delay.**

Grönke et al., *PLOS Genetics* 2010, report an 8-17 hour egg-to-adult developmental delay in *dilp2^1^* homozygous mutants. In our hands, we find that this delay occurs late, after animals reach critical weight, during the L3 larval to pupal transition. To ascertain whether *dilp2^1^* homozygous mutants are delayed earlier, we assayed mouth hook morphology and found no difference compared to controls during early larval stages (S5D,E Figure). This suggests that *dilp2^1^* null mutants are not developmentally delayed from 0 to 48 hours ALH. Furthermore, we assayed EdU incorporation and cortex glial membrane surface area in *dilp2^1^* homozygous mutants at 48 hours ALH (S5B,C Figure). Number of EdU positive neuroblasts and cortex glial membrane surface area was indistinguishable from wildtype control animals. We conclude that Dilp2 is required for neuroblast reactivation and glial membrane growth and that in the absence of Dilp2, other Dilps likely compensate. Future important work will be determining which of the other Dilps compensate.

**3.  Cell type specific rescue experiments could identify the source of Dilp2, test sufficiency and specificity (i.e. would dilp6 overexpression rescue the dilp2 phenotype?).**

When *UAS-dilp2* is expressed in IPCs (*dilp2GAL4*) or in SPG (*moody GAL4*) in *dilp2^1^* homozygous mutants, we found that nutrient-regulated neuroblast reactivation from developmental quiescence was rescued and partially rescued when *UAS-dilp2* is expressed in cortex glia (*NP0577GAL4*) (Figure 6K). When *UAS-dilp6* was expressed in IPCs (*dilp2GAL4*) in *dilp2^1^* homozygous mutants, nutrient-regulated neuroblast reactivation from developmental quiescence was not rescued (Figure 6K). We chose to express *dilp6* in IPCs because this is where Dilp2 is normally expressed at this stage. Dilp2 is not expressed in SPG or other glia at this time. Therefore, we believe the source for neuroblast reactivation is Dilp2 originating from the IPCs.

**While Sousa-Nunes et al. 2011, Chell and Brand 2010 are referenced in the manuscript, a better description of what is already known about Dilp involvement in neuroblast reactivation might help this manuscript to highlight their own contributions, and make it more accessible to non-specialists.**

Thank you for this comment. We have included text to further clarify what is known about Dilp involvement in neuroblast reactivation. The two papers referred to are very high profile publications that have significantly shaped the field's understanding of how neuroblasts reactive. As written in the text, the current dogma in the field is that dietary nutrients stimulate synthesis and secretion of Dilp6 from glia to reactivate neuroblasts. This, of course, could be true in the VNC, but it is certainly not the case in the brain. Using loss of function and rescue experiments, we find that Dilp2 is required and not Dilp6. Importantly, Dilp2 is expressed only in neurosecretory neurons and not glia. Speder and Brand 2018 have since reported as well that Dilp2 is not detected in glia, which is in contrast to Chell and Brand 2010. Therefore, what is significantly different between our work and what is published is the idea that a group of nutrient-regulated neurosecretory neurons located in the brain is what is regulating growth of different cell types in the brain in response to feeding. If neurons are required, it suggests that some type of neural circuit regulation may be in play. Furthermore, two key points of our work that set it apart from published work include: 1) analysis of neuroblast reactivation in each of the *dilp* single mutants and 2) data quantification. We report reactivation based on the number of EdU positive, Dpn positive neuroblasts relative to the total number of Dpn positive neuroblasts. In contrast, Sousa-Nunes et al. 2011 reported "normalized EdU voxels", which is hard to interpret without other cell-specific markers since glia and trachea as well as neuroblast daughters enter S phase and replicate their DNA. Only through a single mutant analysis, can the Dilp be identified that is required for neuroblast reactivation and only through counting the number of EdU positive, Dpn positive neuroblasts can reactivation be quantified.

**The details of the methodology are sufficient to allow the experiments to be reproduced.  
Minor points: 
    1.  In the second block of results “Nutrient dependent growth …”
    - When levels of PI3-kinase activity were reduced in neuroblasts (worGAL4, UAS dp60), neuroblast EdU incorporation was reduced and neuroblast size reduced as previously reported (Figure 2B and S2A) → It would help if the reader were referred to compare S2A to Figure 1J for the Edu incorporation reduction**

done

**- When levels of PI3-kinase activity were reduced in all glia (repoGAL4, UAS-dp60), EdU incorporation was essentially absent in glia and membrane surface area reduced (Figure 2C and S2B,C). → It would help if the reader were referred to compare S2B to Figure 1P for glia membrane surface area reduction. It is unclear how the information that S2C provides fits here**

done

**- Reduction of PI3-kinase activity in trachea (btlGAL4, UAS-dp60), eliminated EdU incorporation and tracheal surface area and branching were reduced (Figure 2D and S2D). → It would help if the reader were referred to compare S2D to Figure 1V for tracheal phenotypes.**

done

**EdU experiments mentioned above are not shown for either glia or trachea, the way it is written makes the reader expect the data in the referred figures. It would be desirable to see these data in the supplementary figure.**

They are in S1 figure H and M and quantified in I and N.

**- Figure 2N, O: The Figure panels in do not match the figure legend.**

Thank you! **2. Discussion 
    - PVF was meant to mean PVR? Please spell out acronym.**

PVR (PDGFR orthologue) ligands **3. Figure legend 6
    - (H) One-way ANOVA with Tukey’s post hoc analysis and (K) Student’s two-tailed t test → Is K supposed to be L?**

Thank you. **4. Figure 7
    - glia (repoGAL4,UAS-mCD8:GFP) at the top of panel A → does not match the mCD8:RFP labeling indicated in panel A or the genotype of these animals as specified in S5 table.**

Thank you. **Authors' responses to Reviewer 4's comments:**

**Reviewer #4: The authors use a series of cell specific alterations of the PI3K pathway to define both autonomous and non-autonomous relationships between neuroblasts, glia, and trachea in regulating their growth. The paper is well written and the experiments are elegant. Overall, the findings of the manuscript provide new and detailed insights into the crosstalk between cells in the brain that are required for proper growth of the brain in response to nutrition. My specific comments can be found below.**

**To determine whether the gial have undergone endo-reduplication the authors could DAPI quantify their nuclei.**

Yes, we could do this, but decided not too because in the end neuroblasts still reactivate in animals expressing *dupRNAi* in glia, which blocks their DNA synthesis (no EdU positive glia) and thus endoreplication. We conclude that the presence or absence of glia endoreplication does not alter neuroblast reactivation profiles.

**Do trachea undergo endo-reduplication?**

This is likely, since it has been published that trachea endoreplicate at other developmental stages. In addition, the tracheal nuclei are quite large in size at 24 hours ALH, which is consistent with an increase in ploidy.

**It would be helpful at the end of the results section relevant to Figure 1 to summarize the specific changes that occur in response to feeding and their relative timing.**

At the end of section 1, we have the following conclusion statement in the text: "we conclude that growth (S phase entry and size) of neuroblasts, glia, and trachea is nutrient-regulated. Moreover, growth of all three cell types occurs continuously and concomitantly, raising the possibility that nutrient-sensing pathways coordinate growth among different cell types".

**How does NB diameter in sucrose fed animals, compare to worGal4 UAS-dp60?**

This data is presented in the manuscript: Please see S1F and S2B.

**How does glial membrane surface area in sucrose fed animals, compare to repoGal4 UAS-dp60?**

This data is presented in the manuscript: Please see Figure 1L and S2D Figure.

**How does tracheal surface area in sucrose fed animals, compare to btlGal4 UAS-dp60?**

This data is presented in the manuscript: Please see Figure 1R and S2F Figure.

**Why do the authors think that glial membrane growth reduction is only modest after inactivation of the PI3 kinase pathway in NBs?**

We were very excited to see that glial membrane growth is reduced when PI3-kinase levels are reduced in neuroblasts. This suggests that somehow reactivated neuroblasts may induce or stimulate growth of glia. The mechanism remains unclear, but is an area we are actively investigating.

**Do they think there are other signals or do they think the expression of dp60 is not strong enough to inactivate the PI3 Kinase pathway?**

Expression of dp60 results in strong reductions in levels of PI3-kinase activity in all cell types that we have assayed to date.

**Why do the authors think that NB incorporation of EdU is only modest after inactivation of the PI3 kinase pathway in trachea? Do they think there are other signals or do they think the expression of dp60 is not strong enough to inactivate the PI3 Kinase pathway?** Yes, we think there are other factors which we added to our discussion.

**What happens to NB size after inactivation of the PI3 kinase pathway in trachea?**

We expect that neuroblast size would be somewhat smaller, however this experiment has not been done.

**A table that summarizes the outcome of the experiments described in Figure 2 would be helpful.**

We have moved the summary cartoon from supplemental figure 2L to Figure 2L.

**“Somewhat surprisingly, neuroblast EdU incorporation in dilp6 null mutants was not changed ...”
Why is this surprising?**

This is surprising because of previously published work reporting that Dilp6 regulates neuroblast reactivation from quiescence. We find that this is not the case in the brain.

**Why is there an affect on NB incorporation of EdU in dilp1 and in dilp7 mutants? In other words, where would the authors put dilp1 and dilp7 relative to dilp2 mechanistically or at least genetically?**

Great question. We do not know why *dilp1* and *dilp7* mutants also have reductions in neuroblast reactivation. Importantly, loss of Dilp2 has the largest effect on neuroblast reactivation. One possibility is that Dilp2 protein levels regulate both Dilp1 and Dilp7.

**Can cell specific activation of the PI3 kinase pathway in one cell type rescue non-autonomously phenotypes seen in other cell types in animals mutant for dilps, or fed sucrose?**

Again, these are great questions and could be areas for future investigation.

**"Trachea allow for oxygen exchange, but are also important sources of cell signaling molecules, including EGF-R and FGF-R.” What are EGF-R and FGF-R? Do they mean the ligands or the receptors?**

Thank you for catching our mistake. We meant to say EGF and FGF, the ligands.
